# When do nudges undermine voluntary consent?

**DOI:** 10.1007/s11098-021-01644-x

**Published:** 2021-05-04

**Authors:** Maximilian Kiener

**Affiliations:** Faculty of Philosophy, The Queen’s College, University of Oxford, High Street, Oxford, OX1 4AW UK

**Keywords:** Nudging, Voluntary consent, Transparency, Resistibility, Rationality, Interpersonal justification

## Abstract

The permissibility of nudging in public policy is often assessed in terms of the conditions of transparency, rationality, and easy resistibility. This debate has produced important resources for any ethical inquiry into nudging, but it has also failed to focus sufficiently on a different yet very important question, namely: when do nudges undermine a patient’s voluntary consent to a medical procedure? In this paper, I take on this further question and, more precisely, I ask to which extent the three conditions of transparency, rationality, and easy resistibility can be applied to the assessment of voluntary consent too. After presenting two examples, designed to put pressure on these three conditions, I show that, suitably modified, the three conditions can remain significant in the assessment of voluntary consent as well. However, the needed modifications are very substantial and result in a rather complicated view. To propose a tidier solution, I argue that nudging undermines voluntary consent if and only if it cannot be ‘interpersonally justified’ to the patient. I use the three modified conditions to motivate the idea of interpersonal justification and also to further specify the principles it involves. My resulting view is especially attractive because it builds on already existing insights from the debate on nudging, updates those insights with an eye to medical consent, and finally unites them in an elegant and simple framework.

**Introduction**

If you inform someone that a medical operation has a 10% chance of failure, they are significantly less likely to consent to it, than if you inform them that it has a 90% chance of success. But why? After all, the two statements are logically equivalent. The answer is that humans are far from perfectly rational. Many of our decisions are based on biases, imperfect heuristics, and mental shortcuts. In the example at hand, it is our human ‘loss aversion’ that makes us less likely to choose something when it is presented in terms of harm rather than benefit (Dean et al., 2017; Kahneman & Tversky, 1972; McKenzie et al., 2006).

Knowing about these features of human decision-making opens up vast opportunities to influence people’s behaviour. Simply by harnessing certain psychological mechanisms one can significantly affect what people do. Thaler and Sunstein famously called this type of influence ‘nudging’ (Thaler & Sunstein, 2009, p. 6), described its huge potential in public policy, and triggered a detailed ethical discussion about its permissible use.

In this paper, I will discuss an under-explored perspective in the ethics of nudging. I will focus on physicians nudging individual patients into consenting to medical procedures rather than on the state nudging its citizens as part of public policy. Moreover, I will discuss whether and, if so, when nudges vitiate a patient’s consent to a medical procedure rather than when nudging is (im)permissible more generally. Given the importance that nudging can have in individual medical encounters as well as the importance of consent for the protection of patient autonomy, the relationship of nudging to medical consent warrants attentions.

I will proceed as follows. In Sect. 2, I will outline the three main conditions for permissible nudging, as developed in the more general literature on nudging: easy resistibility, transparency, and rationality. In Sect. 3, I will ask to what extent these conditions of permissible nudging can be transferred to the assessment of consent too. In other words, I will ask to what extent we can use a *general* theory of nudging, based on these three conditions, to model a *specific* theory of nudging, tasked to explain the validity of medical consent. To do so, I will first devise two examples, designed to put pressure on the three conditions and thereby help us to extract from them what is relevant for consent. In Sect. 4, I will then suggest that the significance of the three conditions for consent is best understood within a framework of *interpersonal justification.* Finally, in Sect. 5, I will conclude and summarise my results. Before I proceed, however, a few clarifications are in order.

Firstly, I will assume that when an influence vitiates consent–in other words, invalidates it–consent no longer renders the performance of a medical procedure permissible. More specifically, without valid consent, performing a medical procedure would wrong the patient, e.g. by amounting to battery, assault, or negligence. In such cases, if the patient does consent, her consent is no longer a sufficiently valuable expression of their autonomy or an effective moral authorisation of the medical procedure at hand.

Secondly, I will focus on only one condition of valid consent, namely *voluntariness*, i.e. the condition that requires a consenter’s decision to be sufficiently free from external control, e.g. coercion, and potentially also from internal impediments such as addiction. Voluntariness itself is a *necessary* condition of valid consent and only provides sufficient conditions for valid consent together with the requirements of competence (i.e. the possession of certain mental capacities) and information (i.e. being informed about relevant details of a medical procedure). To restrict my focus on voluntariness, I will assume that, in all my examples, the person influenced is competent and also sufficiently informed about the medical details of the procedure at hand. Thus, if consent is invalid in my examples, it will always be because it is not voluntary.

Finally, I will rely on a rather wide notion of ‘nudging’, or what I will also call ‘non-persuasive influences’. What I have in mind is close to what Beauchamp and Childress (2013) call ‘manipulation’ or what Blumenthal-Barby (2012) calls ‘non-argumentative’ influences. I will assume that these influences are characterised, negatively speaking, by the fact that they are *neither* rational persuasion *nor* coercion and, positively speaking, by the fact that they trigger certain psychological mechanisms such as biases, heuristics, and mental shortcuts. I consider this wide approach important to capture the great variety of non-persuasive influences that exist and avoid cutting the debate short by a definitional stop.

## The conditions of easy resistibility, transparency, and rationality

The main ethical concern about permissible nudging has been whether nudging undermines the influencee’s autonomy. Whereas some deny that it does by pointing out how nudges can preserve substantial freedom of choice (Saghai, 2013), others are more sceptical and claim that the influencee’s decision-making will often be subverted (Ploug & Holm, 2015). However, most scholars neither fully reject nor embrace nudging. Rather, they claim that nudging is permissible (and autonomy-preserving) under certain specific conditions. Amidst the numerous proposals, the debate has moved towards a set of three key conditions: easily resistibility, transparency, and rationality.

### Easy resistibility

Thaler and Sunstein claimed that nudges “must be easy and cheap to avoid” (Thaler & Sunstein, 2009, p. 6), thereby providing the basis of what has now been developed into a two-part condition of easy resistibility. There is firstly a straightforward or *resilience-based* interpretation of easy resistibility: a nudge is resistible if and only if the person influenced does not experience psychological pressure beyond a certain low threshold and, therefore, acting against this influence does not require special fortitude, resilience, or other mental effort. Secondly, scholars also proposed a *skill-based* interpretation: a nudge is easily resistible on this understanding if and only if one does not need any special psychological skills to resist it (Saghai, 2013). This interpretation is relevant because, on some occasions, resisting a nudge requires psychological expertise on how such a nudge works and how it could be resisted, e.g. in cases of subliminal advertising. Whereas some thought that this requirement of easy resistibility can indeed be met by some or even many nudges (Blumenthal-Barby, 2012; Engelen, 2019a, 2019b; Saghai, 2013), others were more sceptical about nudges ever being easily resistible in the relevant sense (Bovens, 2009, pp. 209-210; Hausman & Welch, 2010, p. 128; Wilkinson, 2013, p. 345). Yet, on both sides of the debate, people accepted easy resistibility as condition of permissible nudging.

### Transparency

In addition, many emphasised the significance of transparency. For instance, Hansen and Jespersen claim that “the distinction between transparent and non-transparent nudges (…) serves as a basis for distinguishing the manipulative use of nudges from other types of uses” (Hansen & Jespersen, 2013, p. 6); and Bovens even argues that “every Nudge should be such that it is in principle possible for everyone who is watchful to unmask the manipulation” (Bovens, 2009, p. 217). Admittedly, there is still controversy over what exact type of transparency is required (e.g. transparency about every particular nudge, transparency about nudging being generally practiced, or transparency about the intention behind the nudging) and also about whether nudging can ever be sufficiently transparent. Yet, both proponents of nudging (Blumenthal-Barby, 2012, pp. 347, 357; Engelen, 2019a, p. 52; Ivanković & Engelen, 2019; Schmidt, 2019, p. 533) and critics of nudging (Calo, 2013, p. 795; Dubov, 2015, p. 497; Greenspan, 2003, p. 163; Grüne-Yanoff & Hertwig, 2016, p. 177; Hausman & Welch, 2010; Wilkinson, 2013) accept some form of transparency as a condition of permissible nudging.

### Rationality

Finally, scholars have argued for a condition of rationality. Schmidt argues that “nudging not only is compatible with rational agency but can even support it” (Schmidt, 2019, p. 511), whereas Conly criticises nudging precisely on the grounds that when we nudge other people, “[r]ather than regarding people as generally capable of making good choices, we outmaneuver them by appealing to their irrationality” (Conly, 2013, p. 30). Most scholars have focused on a condition of *process* rationality rather than *outcome* rationality. A person demonstrates process rationality if and only if the “decision-making procedure should yield accurate results somewhat reliably and not just accidentally” (Schmidt, 2019, p. 512). By contrast, a person demonstrates outcome rationality if and only if, as a matter of fact, he ends up doing what a rational person would do. Whether the process leading to that result was rational too is immaterial to outcome rationality. As in the case of transparency, the correct view on rationality and its relation to nudging has remained controversial. Yet, both proponents of nudging (Cohen, 2013, p. 5; Engelen, 2019b; Hanna, 2015, pp. 622-624; Hansen & Jespersen, 2013, pp. 14-15; Houk, DiSilvestro, & Jensen, 2016, p. 21; Schmidt, 2019, p. 511) and critics of nudging (Conly, 2013, p. 30; Grüne-Yanoff, 2012, pp. 636-637; Hausman & Welch, 2010, p. 134; Ploug & Holm, 2015, pp. 34-37) accept some form of a rationality requirement as a condition of permissible nudging, i.e. a requirement that, roughly, nudging should neither subvert a person’s procedural rationality nor exploit already existing irrationality.

These debates on easy resistibility, transparency, and rationality have provided rich resources for any ethical inquiry of nudging. However, scholars have mostly focused on public policy and the general permissibility of nudging, giving short shrift to the more specific question about how nudging affects the validity of people’s medical consent. So, to what extent can the three conditions help us to assess the validity or, more specifically, the voluntariness of medical consent too?

## The three conditions applied to questions of valid consent

To test whether the three conditions can inform judgments about valid consent, I will devise two cases in this section, designed to put pressure on the three conditions. The first case is based on a situation from real clinical practice (Faden & Faden, 1977) and suggests that the three conditions are not necessary to guarantee voluntary consent, whereas the second case is fictitious and suggests that the three conditions are not sufficient either. After presenting both cases, I will ask how a proponent of the three conditions could reply to such criticism.

### Case 1: Racial Bias

Betty is diagnosed with early-stage cervical cancer. Her physician, who happens to be black, recommends surgery, which would almost certainly cure her. Yet, Betty refuses and says, ‘anyone knows that people with cancer are sick, feel bad, and lose weight’ and adds that, as a matter of fact, she feels very well. She continues to believe that she is healthy despite clear evidence to the contrary. Betty’s disbelief in the diagnosis is further reinforced by a strong racial bias that prevents her from trusting a black doctor.

In this case, Betty relies on a ‘representative heuristic’: since she does not look and feel like the typical ill patient, she does not believe that she is ill (Krawczyk & Rachubik, 2019). Moreover, Betty is subject to a severe form of a ‘confirmation bias’, which means “that information is searched for, interpreted, and remembered in such a way that it systematically impedes the possibility that the hypothesis could be rejected” (Oswald & Grosjean, 2004, p. 80). Betty’s ‘hypothesis’ is a deep racial bias, i.e. the view that black people are neither intelligent nor trustworthy on medical matters. To confirm this view, she interprets her doctor’s statement as false and prioritises her own feeling of being healthy. Finally, Betty may even have been subject to what has been described as the ‘backfire effect’: the more her physician presents evidence contrary to what she believes, the more she clings on to her own mistaken beliefs (Nyhan & Reifler, 2013).

So far, the story is true. But now, let us go beyond the facts and stipulate that Betty has a second, even stronger confirmation bias. Suppose Betty has the firm belief that white men belonging to her religious community are almost infallible. As a result, and to confirm her high opinion of these white men, Betty will consider statements from them always true and authoritative.

Suppose Betty’s physician knows this and therefore asks a nurse, who is a white man and belongs to Betty’s religious community, to tell Betty that she needs to take the medical evidence very seriously, but nothing more. The nurse should only talk about the significance of the evidence and make clear that, based on *this* information, Betty should then make her own decision about whether to undergo surgery. Here, the nurse triggers Betty’s second confirmation bias, silences the effects of the previous representative heuristic, and thereby exerts a clear non-persuasive influence. Yet, *none* of the aforementioned three conditions would be satisfied in this case.

*Transparency:* Betty is not at all aware that the medical team arranged the situation to exploit her confirmation bias and make her appreciate the medical evidence.

*Easy Resistibility:* The lack of transparency prevents Betty from becoming aware of the influence and triggering her propensity to act against it. Moreover, Betty is so indoctrinated and biased that she would believe or do almost anything that the nurse tells her.

*Rationality:* Betty lacks process rationality: her deliberation is not a reliable procedure by which to arrive at a rational decision. Even on the least demanding accounts of process-rationality, i.e. the views claiming that the “decision-making procedure should yield accurate results *somewhat reliably* and not just accidentally” (Schmidt, 2019, p. 521/emphasis added), Betty’s deliberation will be procedurally irrational. Trusting a person because of their sex, skin colour, and membership of a religious community is not at all a reliable procedure in medical matters. It is only in these manipulated circumstances that her bias will lead to an acceptable result. Therefore, even if Betty displays outcome rationality, i.e. appreciating the evidence is what a rational person would do, the nurse’s influence induced a specific kind of (procedural) irrationality in a major part of her deliberation.

Hence, if one relies on the three main conditions from the literature, at least in the general versions I referred to, the influence on Betty will come out as clearly impermissible; and if these conditions are also applied to determine the validity of consent, the influence on Betty will have to be seen as vitiating her consent too. However, this conclusion is too hasty. We should distinguish at least two variants of *Racial Bias.*

In variant 1, and contrary to what the medical team intended, the impact of the nurse’s influence does not stop at making Betty appreciate the evidence. Betty thinks that the nurse also required her to consent. As a result, Betty completely defers to the perceived authority of the nurse and consents to the procedure without deliberating herself. Betty’s decision may be *in accordance with* the available evidence but not *because of* the evidence. In such a case, I concede, Betty’s consent may be non-autonomous and invalid.

However, the situation could be different. Suppose, in variant 2, and aligning with what the medical team intended, the impact of the nurse’s influence *does* stop at making Betty appreciate the evidence. Betty realises that appreciating the evidence is all the nurse wants her do and that she is free to make her own decision about whether to consent. In fact, after being nudged into considering the evidence in this non-transparent, irresistible and irrationality-exploiting way, Betty suddenly sees that she has missed something extremely important, realises how stubborn she has been, and is now determined to base her decision on the available medical facts. Thus, the nurse’s influence shaped Betty’s perception of the situation only up to this point of enlightenment. From there onwards, the nurse’s influence loses its grip on Betty’s decision-making and Betty is solely guided by her recognition of the available evidence. As a result, when Betty eventually consents, she consents not only *in accordance with* the evidence (as in variant 1) but also *because of* the evidence.

Thus, the nurse’s influence can no longer sufficiently explain why Betty consented. To explain Betty’s consent, we need to refer, unlike in variant 1, to Betty’s own deliberation about the medical facts and how surgery will cure her disease. Exploiting Betty’s confirmation bias only enabled her to appreciate critical evidence but left her free to make any decision she wanted. It enabled Betty to deliberate over how to protect her values and interests but did not unduly control her decision-making about whether to consent. For these reasons, Betty’s consent is still a valuable expression of her autonomy and the subsequent performance of the surgery will not amount to battery or assault. But if so, consent must have been voluntary and valid too, despite the fact that the nurse’s influence, which pushed Betty into focusing on a specific set of considerations and without which Betty would not have consented, was still non-transparent, irresistible, and irrationality-exploiting.^1^

Thus, the three conditions, if applied to consent, deliver the wrong result: an influence can violate them all, yet still not vitiate consent. (From here onwards, I will only refer to variant 2 of *Racial Bias.*)

However, one may object that Betty was not nudged in the relevant sense after all. She was nudged into *considering the evidence* but not nudged into *consenting,* and it is only nudging into consenting that is relevant here. This objection makes an important point. There is indeed a relevant difference between influencing people into considering evidence and influencing people into consenting, and I will address this point explicitly in due course. However, we should not discard the influence on Betty as irrelevant at this early point in our inquiry, both because a person’s perception of the evidence is an important part in her motivation to consent and because some nudges into considering evidence (as opposed to nudges into consenting) can invalidate consent. For instance, suppose a physician nudges his patient into considering certain pieces of the evidence by making them very salient to her, while ensuring that other evidence is less salient. In this case, the physician would also not directly nudge the patient into consenting but rather only nudge her into considering the evidence in a certain way. Yet, the nudge would not be unproblematic and would be capable of invalidating subsequent consent.^2^ Hence, the nudge on Betty remains within the ambit of influences that a satisfactory view has to account for.

### Case 2: TV Bully

Alan is publicly known for claiming that people are morally obliged to serve as volunteers in clinical research. Yet, Alan himself has never participated in a trial himself because he is highly averse to undergoing medical procedures. Clinical investigator Carl harbours a grudge against Alan for that reason and wants to end Alan’s (as he sees it) hypocritical behaviour. To do so, Carl decides to surprise Alan during a TV interview. Unbeknownst to Alan, Carl arranges matters so that they will both appear on a particular live show. When the moment comes, Carl confronts Alan in front of the audience and asks him to participate in his clinical trial, a situation which will likely cause Alan embarrassment if he declines. Under the heat of the studio lights and the audience’s expectations, Alan declares that he will participate.

Carl employs a number of non-persuasive influences. To begin with, he exploits what is called the spotlight effect, i.e. people’s tendency to overestimate how much attention they receive from others (Gilovich, 2000), by asking Alan *during a TV interview*. Moreover, Carl also relies on the hot-cold empathy gap in Alan, i.e. the tendency to underestimate how much one’s decision in a situation of agitation differs from what it would be if made with a relaxed state of mind (Loewenstein, 2005), by creating a situation of surprise and potential embarrassment and asking Alan to make an immediate decision during the interview. In addition, Carl plays on the affect heuristic*,* i.e. people’s tendency to act in ways that produce pleasant rather than unpleasant feelings (Barnhill, 2014, p. 56; Slovic, Finucane, Peters, & MacGregor, 2007), by giving him the opportunity to win the audience’s approval rather than their disappointment.

Carl’s various non-persuasive influences make Alan consent to participate in the trial. Note, however, that Carl does not change the incentive structure. He does not make not-consenting to participating in the trial more costly for Alan because the public, let us assume, would have expected Alan to make a decision about his own trial participation in the coming weeks anyway. Carl simply pushed Alan in a carefully-crafted environment designed to nudge him into consenting.

So understood, this case can satisfy the three aforementioned conditions. *Transparency.* Let us assume that Alan knows Carl very well. He immediately recognises what Carl is doing when they both meet on TV, as well as which exact influences Carl is employing. Hence, Carl’s influences are all fully token transparent, to use Boven’s expression, and such token transparency seems to be *the* or at least *one* of the most demanding types of transparency discussed in the literature on nudging.

*Rationality.* Although the array of Carl’s non-persuasive influences will have an a-rational impact on Alan’s decision-making, Alan is aware of them, reflects on whether to follow the push of these influences, and thereby arrives at his decision in a way that is a sufficiently reliable procedure for a rational decision. Thus, Alan’s decision-making will still “yield accurate results somewhat reliably and not just accidentally” (Schmidt, 2019, p. 512).

*Easy Resistibility.* But is Carl’s influence really easily resistible? Admittedly, the average person may, in fact, *not* be able to resist an influence like Carl’s easily as doing so seems to require great psychological effort, given how much pressure one might feel during a public TV interview. However, the situation could be different for Alan. Suppose Alan is a seasoned interviewee who knows how to dodge questions. Although Alan consents, perhaps partly because he is tired after a long day and does not want to displease his opponent Carl once again, he still has the ability to manoeuvre his way out of those nudges relatively easily and decline to participate or refuse to make a decision during the interview.

Hence, in this scenario, the three conditions are all satisfied and, therefore, the traditional view suggests that Carl’s nudging was permissible. Moreover, if applied to consent, the traditional view also suggests that Alan’s consent remains perfectly valid. But these judgments are wrong. To begin with, Carl still *illegitimately influenced* Alan. Carl *influenced* Alan by deliberately crafting the context in which Alan eventually decided to consent and this context, viz. the range of non-persuasive influences, happened to play a key causal role in the events leading to Alan’s consent. Such influence is *illegitimate* because, firstly, it still amounts to manipulation in the morally pejorative sense which Noggle and others think is characteristic of manipulation more generally, i.e. it decreases the quality of the target’s decision-making (Noggle, 1996, 2020).^3^ Carl decreases the quality of Alan’s decision-making by (i) subjecting Alan to a-rational influences which he would otherwise be free from and the sole purpose of which is to promote Carl’s interests over Alan’s, and (ii) by cutting short Alan’s process of deliberation: in the absence of Carl’s intervention, Alan would have taken more time to think about the matter and may have figured out an even better solution to avoid participating without incurring public embarrassment.

Secondly, Carl’s influence is also illegitimate because it is essentially a trap. Carl went behind Alan’s back to arrange the situation so as to surprise Alan live on TV. Although the nudges themselves were fully token transparent when they happened, Carl did not tell Alan about his intentions before the interview and tricked him into a situation designed to make him consent.

Finally, Carl’s influence is illegitimate because he violates a professional obligation that clinical investigators owe their prospective volunteers. Investigators like Carl are obliged to respect people’s autonomy in making medical decisions, which requires that they promote or at least not sabotage people’s decision-making as Carl did. Particularly in the context of clinical research, where medical procedures hold no medical benefit for those participating in a trial, respect for autonomy is especially important and implies, at a minimum, an especially strong negative duty not to interfere with a person’s autonomous decision-making (Berg & Applebaum, 2001, p. 279; Boddington, 2012, p. 178; Nelson-Marten & Rich, 1999, p. 81; Pellegrino, 1992).

Hence, the traditional view that the three conditions are sufficient to rule out impermissible nudging is mistaken. Whatever else we may think about *TV Bully,* Carl acts wrongly in nudging Alan and is therefore liable to, at least, certain disciplinary sanctions.

But the problem with the traditional view is not just that it fails to identify impermissible nudging. It also fails to identify consent-invalidating nudging. This is because Carl not only illegitimately influences Alan, he also – more specifically - renders Alan’s consent *invalid*, or at least this is what two plausible approaches to consent suggest. To begin with, David Owens claimed that influences invalidate consent when they *wrong* the consenter (Owens, 2007, 2012, chapters 2 and 7). In *TV Bully*, Carl wrongs Alan by violating the negative duty not to sabotage Alan’s decision-making. This is a very significant duty in medical contexts and fundamental to the relationship between clinical investigators and prospective trial participants. Thus, in Owens’s plausible and indeed widely shared view, Alan’s consent is invalid.

Moreover, in his contractual theory, Samuel Pufendorf claimed that consent is invalid if the recipient ‘disqualified’ himself in the process of obtaining that consent (Pufendorf, 1998, Liber III Cap VI: §X-XI).^4^ ‘Disqualification’ means the violation of a norm that is constitutive of the role one occupies in receiving another person’s consent. In *TV Bully,* Carl receives Alan’s consent in his role as a clinical investigator. Yet, in the very process of obtaining that consent, Carl violates norms central to this role, viz. he violates the aforementioned negative duty not to sabotage people’s decision-making and thereby ‘disqualifies’ himself as a recipient of Alan’s consent. Thus, in Pufendorf’s view too, Alan’s consent to Carl is invalid.

Hence, on two plausible views concerning invalid consent, i.e. Owen’s wronging view and Pufendorf’s disqualification view, Carl not only illegitimately influenced Alan, he also invalidated Alan’s consent. Therefore, the three conditions seem to deliver the wrong result again: they can all be satisfied, yet consent is still invalid.

Yet, I admit that, in some contexts outside medicine, consent could be classified as voluntary even if obtained by the means Carl employed. But it seems reasonable to set the threshold higher in the medical context, given the importance we attach to our bodily integrity and the special obligations of medical professionals.

### Potential response to *Racial Bias *and *TV Bully*

Thus, I have now presented two examples designed to put pressure on the three conditions. The first suggested that the three conditions are not necessary to guarantee voluntary consent, whereas the second suggested that they are not sufficient either. However, these two examples are complex and far from the ordinary. For this reason, they are not meant as decisive arguments against the three conditions. I only use them as test cases, designed to reassess the three conditions in certain ways, and as just one element in a larger coherentist picture in favour of my own proposal, which I will present in Sect. 4. So, with this clarification at hand and in order to work towards my own view, let me now ask how someone intending to defend the three conditions in the assessment of voluntary consent could reply to my examples. Can the three conditions be in some way rescued for the assessment of voluntary consent?

#### Transparency

When discussing nudging in public policy, Thaler and Sunstein argued that, at least *hypothetically* speaking, governments must be prepared to justify their nudging practices in public (Thaler & Sunstein, 2009, p. 243ff). Building on this thought, a modified transparency condition might opt for a disjunctive view, requiring an influence to be either actually transparent *or* such that, *hypothetically* speaking, it *could* be made transparent and thereby justified to those affected by it. Thus, to defend transparency, one could introduce *hypothetical* transparency as a further and for the validity of consent sufficient requirement.

This view seems to be promising as a way of accommodating our intuitive response to *Racial Bias:* even though the medical team did not actually make their influence transparent to Betty, they could - hypothetically speaking - later do so and justify it to Betty on the grounds that it assisted Betty in pursuing her own conception of the good. So understood, the influence on Betty satisfies the requirement of (hypothetical) transparency and accounts for my first case *Racial Bias*.

But this response comes at a cost. What matters in the end is not the possibility of making the influence *transparent* but rather whether, in so doing, one could also *justify* it, and in particular justify it *to Betty* and in terms of Betty’s own values. So, it is this (interpersonal) justification, and not transparency, that is doing the actual normative work and determines whether the influence invalidates consent. If anything, transparency could be a *test* (for interpersonal justification): since only suitably justifiable influences could be made transparent to Betty without her feeling legitimate resentment, we can use hypothetical transparency as a test to separate justifiable from unjustifiable influences.

A similar conclusion, namely that it is not transparency that is doing the actual normative work, seems to arise when considering my other example *TV Bully*. Here, Carl’s influences are actually and fully transparent. Yet, such transparency does not speak in favour of Carl’s influence being permissible, or compatible with Alan’s voluntary consent. Here, transparency only *excludes* other potential problems associated with *non*-transparency, e.g. problems of deceit. Thus, in *TV Bully,* transparency seems to be best understood, not as a condition of permissibility of voluntary consent itself, but rather as a way of ensuring that certain other problems do not arise. I therefore conclude that transparency, although still significant, should not be seen as a genuine condition of valid consent. Rather, in its hypothetical form, it is a *test* for justifiability and, its actual form, it *excludes* certain other challenges to valid consent. So understood, we can rescue the significance of transparency while at the same time accounting for the intuitively correct assessment of my two examples.

#### Rationality

But what about rationality? Is there a way to defend it as a condition of influences preserving valid consent? To answer this question, I will first need to outline three striking differences between *Racial Bias* and *TV Bully.*

1. In *Racial Bias,* the influence on Betty targeted her appreciation of the evidence, as opposed to her decision to consent. Thus, the influence may have made Betty’s deliberation about why she should consider the evidence irrational, but it did not make Betty’s subsequent deliberation about whether to consent in any way irrational. After all, she consented for correctly appreciated health reasons. By contrast, the influence on Alan directly targeted his decision to consent rather than his appreciation of already existing evidence.
2. In *Racial Bias,* the influence did not make Betty’s deliberation worse than it would *otherwise* have been. In any case, Betty would have been subject to the confirmation bias in one way or another. By contrast, in *TV Bully,* Carl’s influences made Alans’s deliberation subject to certain psychological heuristics and biases that would otherwise *not* have affected his decision.
3. In *Racial Bias,* the influence supported Betty in pursuing only her own conception of the good, i.e. by enabling her to make a decision in light of all the relevant facts together with her own individual values. Thus, Betty’s rationality was fully in service of her own ends. By contrast, in *TV Bully,* Alan’s rationality was put to the service of Carl’s ends. The influences changed the situation so that Alan’s procedural rationality would inevitably promote Carl’s goal of trial recruitment too. Thus, in some way, these influences hijacked Alan’s rationality.

On the basis of these three differences, one could modify the rationality requirement so that it will be met in *Racial Bias* and violated in *TV Bully.* This modified rationality requirement would hold that an influence vitiates consent if it (i) controls the decision to consent, as opposed to the appreciation of correct and balanced evidence, (ii) renders the subject’s reasoning (considerably) less rational than it would otherwise have been, and (iii) forces a subject’s procedural rationality into serving other people’s ends at the expense of his own. So understood, the influence on Betty does not violate this requirement, whereas the influences on Alan do. Hence, in this suggested modified version, the rationality requirement can successfully deal with my examples *Racial Bias* and *TV Bully,* and therefore be potentially retained as an actual condition for influences preserving valid consent.

This modified rationality condition is a novel addition to the ethics of nudging. Yet, aspect (ii) has also a possible basis in the existing literature, at least if we classify the various rationality objections against nudging into two different groups.^5^ On the one hand, scholars argued that nudging *subverts* rationality. For instance, it was held that nudging “perverts decision-making” (Wilkinson, 2013, p. 349), “perverts people’s rationality and thus makes them less rational” (Engelen, 2019b, p. 206), or “undercuts people’s rational agency” (Schmidt, 2019, p. 515). I take it that the thought here is that nudging induces forms of irrationality in people’s decision-making that would otherwise not arise. On the other hand, however, scholars also argued that nudging *exploits* irrationality. For instance, Bovens argued that in cases of nudging “some pattern of irrationality is being exploited” (Bovens, 2009, p. 209) and Conly holds that, as nudgers, “[r]ather than regarding people as generally capable of making good choices, we outmaneuver them by appealing to their irrationality” (Conly, 2013, p. 30). I take it that the thought here is, *not* that nudging creates *additional* and otherwise non-existing irrationality, but rather that it exploits, harness, or appeals to already existing irrationality in people’s decision-making. Although many people gloss over this distinction between subverting and exploiting irrationality, it can be drawn and, since it accounts for the needed clause (ii) in my modified rationality requirement, it is highly morally significant.

#### Easy resistibility

Finally, let me test whether the condition of easy resistibility could be defended. At first, this seems very difficult. The condition of easy resistibility did not fare well in my two examples. The influence on Betty was very hard to resist, yet it did not vitiate her consent. By contrast, the influence on Alan was, at least *for him*, easy to resist but still vitiated his consent. Hence, these cases suggest that easy resistibility cannot be made a condition of valid consent.

However, the condition of easy resistibility fares much better in more ordinary cases. For instance, if Alan was not such an experienced interviewee, Carl’s influence would have been hard to resist indeed. Here, the lack of easy resistibility would have tracked Carl’s influence as consent-vitiating. Moreover, a severe threat, e.g. a threat of bodily harm, is often also very hard to resist and a paradigmatic example of an influence that vitiates consent. Thus, *these* cases suggest that easy resistibility can indeed be used when assessing whether a person’s consent is valid.

To account for this mixed result, I suggest that easy resistibility should be seen, not as a *condition* for valid consent, but rather as an *imperfect proxy* for something else that is such a condition, namely the absence of illegitimate control. Let me elaborate.

I understand ‘illegitimate control’ as the exertion of pressure on the consenter that deprives him or her of a fair opportunity to make a free decision. Here are a few examples: someone illegitimately controls the consenter’s decision-making and removes a fair opportunity when he renders not consenting costly in a significant way (e.g. by imposing a penalty as in cases of threats), when he exploits the consenter’s already existing decisional weakness (e.g. by harnessing the consenter’s situation of tiredness, anxiety, agitation, or other similar state) or even creates such a weakness in the first place, when he actively diminishes the consenter’s *emotional* capacities for decision-making (e.g. by shaming him for his body in order to obtain consent to cosmetic surgery) (Eyal, 2014), when he actively diminishes the consenter’s *cognitive* capacities for decision-making (e.g. by distracting the consenter or appealing to phobic tendencies to obtain consent) (Noggle, 2018, p. 169), or when he excludes the consenter from important parts of the decision-making (e.g. by working on the consenter’s unconscious psychological tendencies, as in certain cases of ‘priming’).

This list of examples of illegitimate control is not exhaustive, yet it still elucidates what illegitimate control comprises. In all of these examples, the influence from the person receiving consent (i.e. the consentee) wrongs the person giving consent (i.e. the consenter) during the process of obtaining consent. By ‘wronging’ I mean violating a claim that the consenter can make on the conduct of the consentee when the latter obtains his consent. What counts as a wronging therefore depends on the context and, more specifically, on the norms governing the relation between the consenter and consentee. For instance, in the medical context, it is fundamental to the patient-physician relationship that the patient is entitled to respect for his autonomy and promotion of his well-being. Thus, these two principles of respect for autonomy and beneficence can explain why a physician wrongs the patient when he exerts certain influences, e.g. penalises, shames, or distracts the patient. I will further elaborate on the relevant principles and claims in the medical context when developing my own view in Sect. 4. At this point, it suffices to leave the elaboration of ‘illegitimate control’ at this general level, i.e. as the exertion of pressure that violates a claim that the consenter can make on the conduct of the consentee.

So understood, illegitimate control tracks consent-vitiating influences: in Betty’s case there was no illegitimate control: she was not deprived of fair opportunity to make a free decision about whether to consent, but only enabled to make a decision based on her own value and on the facts. If anything, Betty’s standing in the decision-making process has been strengthened. By contrast, in Alan’s case, there was illegitimate control: independent of the pressure Alan actually experienced, Carl set up a trap for Alan, made Alan’s decision-making worse than it would otherwise have been, and violated an important professional obligation that he has *qua* clinical investigator. Finally, there is also clear illegitimate control in cases of threats, as threats penalise non-compliance and remove options to which the consenter is otherwise entitled.

However, even if illegitimate control is doing the actual normative work in the assessment of valid consent, easy resistibility can still serve as a proxy. This is because, *normally,* influences that are not easy to resist are also those that are likely to usurp too much control over another person’s decision-making and thereby exert illegitimate control. Normally, being threatened comes with great pressure, being shamed comes with great pressure, and so on. Yet, the qualification *normally* is important because easy resistibility is only an *imperfect* proxy, as my examples about Betty and Alan show. Hence, the best way to defend the significance of easy resistibility against my two examples is to describe it as an imperfect proxy for illegitimate control.

But note that this conclusion is not restricted to the realm of non-persuasive influences. It can also be supported in the context of other types of influences, e.g. threats and offers. To see this, suppose I offer you one million £ in exchange for your sofa and you really need the money to pay your mortgage. Such an offer is very hard to resist as it requires great resilience or insensitivity to financial gain. Yet, other things being equal, your consent to selling the sofa remains voluntary and valid, precisely because my offer did not illegitimately control your decision-making, i.e. it did not deprive you of a fair opportunity to make a free decision. Contrast this case with another scenario where I threaten to beat you unless you sell the sofa to me. It may be easier for you to resist this threat than to decline my previous offer. Yet, even if this is the case and you happen to be the person who can resist threats more easily than offers, your consent to selling is forced, involuntary, and can be voided; and this is because the threat of physical harm constitutes the kind of illegitimate control that vitiates a person’s consent.^6^ Thus, the distinction between resistibility and illegitimate control is of more general importance and should be part of any inquiry into the voluntariness of consent.^7^ Therefore, I can reach one further final conclusion: even if a clever reader finds a way to deny that Carl’s influence was easily resistible for the seasoned interviewee Alan, this would be a dead-end, because it would commit to the false view that easy resistibility determines whether consent is voluntary.

## The three conditions within a framework of interpersonal justification

Thus, suitably modified, the three conditions can still help us assess voluntary consent. Yet, the required modifications are substantial and lead to a rather complicated solution, including many nuanced distinctions and the downgrading of transparency and resistibility from genuine conditions to a mere test and proxy. In this section, I therefore want to account for my results within a simpler and more elegant approach, namely the following: nudging vitiates medical consent if and only if it cannot be *interpersonally justified*.

### Outline of interpersonal justification

Interpersonal justification, as I will understand it, is an adequate reply to the question “Why is it permissible for *you* (*qua* physician) to obtain consent from *me* (*qua* patient or trial participant) in this way?” To answer this question, the person receiving consent, i.e. the physician or trial investigator, needs to show that their influence on the person giving consent is permissible in light of the claims that the person giving consent can make on them *qua* the person receiving consent. Hence, I focus on the interpersonal justification of an influence *from* the person giving consent *to* the person receiving consent. But I only require hypothetical justification. It is not necessary for the physician to provide an actual answer to the aforementioned question or for the patient actually to ask it.

So understood, interpersonal justification contrasts with justification *all things considered*, which is much broader and requires that something is permissible in light of *all the relevant* moral considerations, and not just the claims that the person giving consent can make on the conduct of the person receiving consent. Moreover, it is worth noting that interpersonal justification is not a trivial consequence of justification all things considered. It is *not* the case that the physician’s conduct will be, firstly, justified on grounds unrelated to the patient and then, only as a consequence, be justifiable to anyone, including the patient. The grounds for interpersonal justification will always be relative to the person consenting and the claims that this person can make on the person receiving consent.

On this basis, actions can be justified interpersonally but unjustified all things considered, and vice versa. For instance, if a woman consents to an abortion, the physician’s performance of it is interpersonally justifiable to the woman, given her consent. However, the woman’s consent cannot determine whether such an abortion is also justified all things considered, i.e. in light of all the relevant facts. After all, the foetus might already be able to live outside the woman’s uterus and thereby make the abortion unjustified all things considered, according to some. Conversely, there may be cases where an action is interpersonally unjustified, but still justified all things considered. Twisting a person’s arm might violate their claim to bodily integrity and thereby be interpersonally unjustiable but still be justified all things considered, e.g. when twisting that arm is necessary to save thousands of lives (Nagel, 1986, p. 176).

The idea of interpersonal justification plays an important role in other philosophical debates. Most importantly, in the debate about contractualism, scholars asked what we *owe to each other* (Scanlon, 1998), thereby also alluding to the aspect of what can be justified *to certain others* rather than justified all things considered. Moreover, philosophers often distinguish between *wrongdoing* and *wronging* (Dougherty, 2015, p. 232; Mayr, 2019, pp. 118-124; Tadros, 2016, p. 201; Thompson, 2004, pp. 333-335), and thereby rely on a similar contrast too: wrongdoing consist in the performance of an action that is impermissible *in light of all the morally relevant facts*, whereas wronging consist in the performance of an action that is impermissibl*e in light of what is owed to a particular person*, e.g. impermissible because it violates a right that one owes to that person*.* Thus, my contrast between interpersonal justification and justification all things considered has a basis in the philosophical literature more generally.

Hence, I am not inventing the idea of interpersonal justification from scratch in this paper. Rather, my novel contribution is to take it from the debate in normative ethics, introduce it to the debate on consent and nudging, and develop it further so that it can inform judgments about the validity of consent.

But note that the idea of applying interpersonal justification to consent does not come out of the blue. My discussion of transparency explicitly highlighted it. Here, it was key that an influence could not only hypothetically be made transparent but also *justified to the person affected*, i.e. *interpersonally justified*. In addition, regarding rationality, I claimed that an influence needs to respect the consenter’s entitlement to pursue their own conception of the good. This entitlement is at least partly a claim of non-interference which the consenter can make on the conduct of others, e.g. the physician. Therefore, respecting this claim is a means to ensure interpersonal justifiability. Finally, regarding easy resistibility, I argued that easy resistibility is a proxy for the absence of illegitimate control and I explained that illegitimate control consists in the violation of claims that the consenter can make on the conduct of the consentee, i.e. those claims that also shape interpersonal justification. Thus, interpersonal justification may be the unifying consideration behind the complicated modifications of the three conditions and lead to a more elegant approach to voluntary consent.

However, to make full use of this idea of interpersonal justification in the assessment of consent, I need to specify it further. What exactly does interpersonal justification require in the context of valid consent? What precisely are the claims that the consenter can make on the conduct of the person receiving consent? And what are the principles that need to be satisfied for an influence to be interpersonally justifiable in a way that preserves the validity of consent?

Fortunately, the modified three conditions can provide further guidance on these questions too. In the next section, I will refer to the three modified conditions in order to, firstly, outline in greater detail what the foundations of interpersonal justification in the context of consent are and, secondly, thereby situate the three modified conditions in a more general framework that features three corresponding principles of interpersonal justification.

### The principles of interpersonal justification in the medical context

To begin with, my discussion of hypothetical transparency seems to point to a principle of trust. By way of explanation, when we trust another, we can neither spell out the precise terms which the trusted person should act on nor completely control or monitor the trusted person’s conduct. In fact, the very point of trust is the reliance on a person’s benevolence in situations where we are unable to control or monitor that person. But as I have explained elsewhere, trust also always comes with the *proviso of hypothetical transparency*:

“Even though a trusting person refrains from ‘the precise spelling out of terms’ (Greenspan 2003, 161) which the trusted person may operate on, trust always comes with the proviso that the trusted party’s relevant actions as well as ‘the motives of the trusted party have to be able to be made transparent to the one who extends trust’ (Ibid.). This proviso is respected only if full transparency did not lead to the trusting person being justified in feeling resentment or insulted.” (Kiener, 2018, p. 193)

Hence, acting in ways that could hypothetically be made transparent is a way of honouring a person’s trust, and therefore the significance of hypothetical transparency, as I explained it earlier, could be accounted for by a wider principle of trust. However, trust not only requires hypothetical transparency. It also requires that the trusted person does not abuse their superior position of power more generally, independently of whether doing so would actually be transparent to the trusting person. Thus, including this further aspect, I suggest the following first principle of interpersonal justification:

#### **Trusted partner**

The medical professional is obliged to honour the consenter’s trust, i.e. he is obliged to act in ways which (i) could be made hypothetically transparent without causing justified resentment in the consenter and (ii) do not abuse a superior position of power relative to the consenter.

So understood, *Trusted Partner* allows influences to remain non-transparent as long as transparency would not lead to justified resentment, and thereby accounts for my conclusion in Betty’s case; but *Trusted Partner* still proscribes even fully transparent influences when they unduly dominate the consenter, i.e. it proscribes Carl’s influence on Alan, where Carl clearly exploited his superior position of power to dominate Alan’s decision. Hence, *Trusted Partner* does not only build on my earlier results on hypothetical transparency but also accounts for the intuitively correct results in my examples. Therefore, we can use it as first pillar in an account of consent and interpersonal justification.

Secondly, in my discussion of the condition of rationality, I claimed that it was key whether an influence forces a person’s rationality into serving of other people’s ends or whether it allows a person to pursue their own ends and conception of the good. Building on this insight and being inspired by John Rawls’s important work on people’s entitlement to pursue a conception of the good, I suggest this second principle of interpersonal justification:

#### **Equal moral standing**

The medical professional is obliged to respect the consenter’s entitlement to form, revise, and rationally pursue a conception of the good.

I call this principle Equal *‘Moral’* Standing because it is about people exercising what Rawls called a *moral power*: the capacity to “form, revise, and rationally pursue a conception of the good” (Rawls, 2005, p. 30). I call it *‘Equal’* Moral Standing because it obliges physicians to acknowledge that patients, despite being in an asymmetrical relationship to physicians in other respects, are still on a par with them regarding the entitlement to pursue a conception of the good. So understood, *Equal Moral Standing* builds on the insights from the discussion of rationality and accounts for my earlier results. It captures the fact that, in Alan’s case, the influences were problematic because Alan’s rationality was pushed into serving other people’s interests, while also capturing my view in Betty’s case that the influence was less problematic because the medical team allowed her to pursue her own values.

Finally, recall my discussion of easy resistibility, where I argued that the psychological impact of an influence alone cannot determine whether an influence vitiates consent. What matters is the presence or absence of illegitimate control. This discussion seems to point to the following third and last principle of interpersonal justification:

#### **Assisted decision-making**

The medical professional is obliged not to illegitimately control but only to assist his patient’s decision-making.

*Assisted Decision-Making* focuses on illegitimate control rather than easy resistibility and thereby builds on my previous conclusions. However, *Assisted Decision-Making* also goes beyond my previous discussion by specifically permitting influences that *assist* a person’s decision-making. To assist a patient’s decision-making, a medical professional can frame medical information in a way conducive to understanding, actively help the patient to clarify views and options, make recommendations, and even push a patient into considering relevant evidence, as in Betty’s case. How much and which assistance is appropriate depends on what a person consents to and the wider circumstances. More serious medical procedures may require greater assistance than less serious procedures. Moreover, in some cases, assistance may require joint deliberation, whereas in others, e.g. emergency cases with little time for a decision, assistance should take the form of a straightforward recommendation. On this basis, *Assisted Decision-Making* identifies the influence on Betty not as illegitimately controlling but rather as assisting, while it marks the influence on Alan as illegitimately controlling. The key features of *Assisted Decision-Making* are that, firstly, it explicitly allows influencing patients in ways conducive to their decision-making, while at the same time setting clear boundaries in doing so; and secondly, it rejects a mere psychological focus and considers influences as exerting illegitimate control even if they are easily resistible, as in the case of the seasoned interviewee Alan.

Thus, the three conditions lead us to the view that an influence is interpersonally justified if and only if it is consistent with the more general principles *Trusted Partner, Equal Moral Standing,* and *Assisted Decision-Making.* Hence, in my view, the person giving consent can claim that the person receiving consent abide by these principles, and if he fails to do so when influencing the person giving consent, his influence will vitiate consent. Admittedly, the three principles overlap with each other in certain respects, but they still all have their distinctive role. *Trusted Partner* accounts especially for the significance of hypothetical transparency, *Equal Moral Standing* for the importance of not forcing people’s rationality into serving other people’s ends, and *Assisted Decision-Making* for the important distinction between resistibility and illegitimate control. On this basis, we arrive at the following hierarchical scheme in the assessment of voluntary consent.
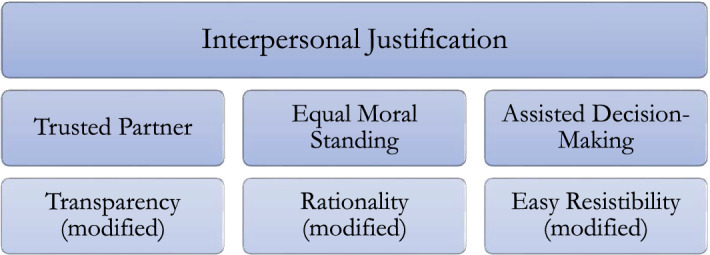


On the top, there is the most general idea of interpersonal justification: nudging undermines voluntary consent if and only if it fails to be interpersonally justifiable to the consenter. On the next level, there are three more specific mid-level principles that explain what interpersonal justification requires, namely that an influence be consistent with the demands of *Trusted Partner, Equal Moral Standing,* and *Assisted Decision-Making.* Finally, at the bottom, there are the modified conditions of transparency, rationality, and easy resistibility, from which I developed the principles at the second level. The three conditions at the bottom are more specific than the mid-level principles at the second level and can provide further guidance in clinical practice. However, a proper assessment of voluntary consent requires that transparency, rationality, and easy resistibility are always interpreted in the light of the idea of interpersonal justification and its more general principles *Trusted Partner, Equal Moral Standing,* and *Assisted Decision-Making.* Hence, by incorporating the three conditions within this framework of interpersonal justification, my hierarchical model can retain the significance of the three conditions in the context of consent.

### Further clarifications and contrast with other views

Having laid out the structure of my own proposal, I will now clarify some of its details, starting with a potential objection. Suppose a physician wants to harm her patient she knows is inclined to buy into an anti-vaxx conspiracy theory. The physician makes the patient focus heavily on the potential side-effects of the vaccine by tapping into his irrational fears via some well-designed nudges, e.g. by exaggerating his fear that governments use vaccines to harm its citizens. In this case, the physician’s nudges may seem to be interpersonally justifiable to that patient because they trigger what are, *by the patient’s own lights*, reasonable grounds for refusing the vaccine. Yet, this appears to be a case of impermissible or even consent-invalidating nudging.^8^

Fortunately, my view is compatible with this intuition and does not imply that influences are interpersonally justifiable if they trigger what patients already accept. This is because interpersonal justification rests on three principles which do not fully depend on the perspective of the patient. In this case at hand, the physician would at least violate *Assisted Decision-Making* as she clearly subverts rather than enhances her patient’s decision-making. After all, the physician would reinforce a false belief as the ground for consent and this is inconsistent with respect for patient autonomy as well as her professional duty to act beneficently. Thus, although interpersonal justification requires specifically focusing on the claims that the consenter has against the consentee, rather than moral norms in general, it does not imply that an individual consenter always knows what his claims against the consentee are or that he always knows when, or can always decide if, something violates such a claim.

But let me vary this example. A physician meets a woman described as a ‘conspiracy theory magnet’. When the woman refuses vaccination on the grounds of conspiracy theories, the physician asks her:

‘Have you considered the possibility that anti-vaccine propaganda could be an attempt by the Russians or the Chinese to weaken the health of the United States population?’^9^

The woman looks shocked, remains silent for a moment, and eventually consents to the vaccination. This case differs from the previous one as the physician makes the patient consent to beneficial and potentially urgently needed medical treatment.

My view offers a nuanced take on this case. Suppose the physician just asked the quoted rhetorical question to make the woman aware that her beliefs actually support a variety of inconsistent conclusions. As a result, the physician’s question makes the woman realise that her beliefs are ill-founded and she consequently stops believing in any of those conspiracy theories. Here, the physician’s intervention is interpersonally justifiable: it enhanced the woman’s decision-making and allowed her to make her own decision in the end.

But alternatively, it could also happen that the rhetorical question makes the woman immediately adopt that other conspiracy theory: she is now fully convinced that the Russians or the Chinese try to weaken the health of the United States population, and therefore consents. Let us assume that the physician does not object to this false belief but on the contrary reinforces it, e.g. by making a certain website that endorse this conspiracy theory very salient to her or even by pretending to believe it himself. In this scenario, vaccination may still be in the best interests of the woman. Yet, according to my view, this intervention qualifies as impermissible and consent-invalidating, because the nudges fail to treat the woman as an equal in the decision-making process (they steer her towards a decision without strengthening her standing in the process), and encourage the woman to base her decision on a false belief, thereby sabotaging her decision-making. This appears to lead to a violation of *Equal Moral Standing* and *Assisted Decision-Making,* which would render the nudge interpersonally un-justifiable in my view.^10^

My position on this case sets my view further apart from other accounts, in particular from those focusing on a person’s best interests. For instance, Gorin *et al.* argue that “the best-interest standard (…) offers a robust justification for the use of nudges” (Gorin et al., 2017, p. 33), at least in situations where a physician cannot mitigate a patient’s biases through an open conversation. Gorin *et al.* explain that ‘best interests’ must be understood objectively, which means that they “must appeal to an ethical and professional standard, not to patients’ preferences” (Gorin et al., 2017, p. 33). Finally, they add that “[r]espect for patient autonomy does not (…) require that all patient preferences be honored, regardless of how informed or competent they are” (Gorin et al., 2017, p. 36). Although Gorin *et al.* do not address my example, they appear to imply that the anti-vaxx nudge is permissible in both scenarios. After all, it was necessary to make the woman consent to what is in her best interests.

However, it seems to be a weakness of Gorin *et al.*’s view that they are likely to gloss over the differences between the two scenarios and only focus on the woman’s best interests. After all, when inducing false beliefs and taking advantage of the woman’s cognitive shortcomings, the physician clearly acts wrongly, however beneficial the subsequent vaccination may be. For this reason, my proposal, which offers a more nuanced view on the two scenarios, seems to be superior.

Moreover, Gorin *et al.’*s view, unlike mine, seems to conflict with two plausible views in the more general debate on consent, which I referred to earlier, i.e. Owen’s wronging view and Pufendorf’s disqualification view. This is because, in the scenario where the physician induces a false belief, she wrongs the patient and, by being untruthful, also violates an important professional norm. Hence the physician cannot obtain valid consent in the way she aims to. Yet, Gorin et al.*’*s view might suggest otherwise and thereby position itself against two more general and very plausible views about consent.

However, over and above these reasons to prefer my proposal over Gorin et al.’s*,* there are also two more general differences between our views. Firstly, insofar as best interests are relevant in the evaluation of nudging at all, I reject Gorin et al.’s objective standard. I here side with Holm, who argued that what is best for one person may not be best, or even good, for another person, because people’s values and preferences differ (Holm, 2017). My own view, especially its principle *Equal Moral Standing,* requires that we respect individual people’s entitlement to pursue various conceptions of the good and it thereby proscribes the imposition of an objective standard.

Secondly, I disagree with Gorin et al. that one may permissibly nudge a patient into what is in his best interests (however conceived) once that patient’s bias cannot be mitigated via rational persuasion. In fact, Gorin et al. seem to neglect that, in many cases, nudges can be used not only to steer people but also to enhance their decision-making (Gelfand, 2016; Ploug & Holm, 2015, p. 35). For instance, in order to prevent a patient’s cognitive and emotional overload from determining her decision, one could employ a *Cooling Off Period,* i.e. nudge a person into making a decision at a later point.^11^ Moreover, some nudges can enhance decision-making by triggering affect heuristics, i.e. people’s tendency to be influenced by their emotions in response to an interaction. For example, a physician could deliberately speak in a slow and calm voice to reduce cognitive and emotional overload (Hanna, 2015). Whereas Gorin et al. neglect these options, my view, emphasising that physicians *assist* patient decision-making, prioritises them. As a result, my view not only seems to offer greater respect for people as autonomous decision-makers than Gorin et al. do, it also highlights another way to promote a person’s best interests, namely by using decision-enhancing nudges to help people make better decisions on their own and thereby potentially promote their best interests most effectively.^12^

## Conclusion

The permissibility of nudging in public policy is often assessed in terms of the conditions of transparency, rationality, and easy resistibility. I credited this debate for producing important resources for any ethical inquiry into nudging, but also pointed out that scholars have not directly focused on a different and very important question, namely when nudges vitiate medical consent. I then presented two cases designed to show that the three conditions are neither necessary nor sufficient for voluntary consent to a medical procedure. I conceded that, suitably modified, the three conditions remain significant in the assessment of voluntary consent, but these modifications result in a rather complicated solution. I have proposed a tidier solution, namely, that an influence vitiates medical consent if and only if it cannot be *interpersonally justified*. I used the three modified conditions not only to motivate the idea of interpersonal justification but also to further specify the principles it involves. This resulting hierarchical view is especially attractive because it builds on already existing insights from the debate on nudging, it updates those insights with an eye to medical consent, and it finally unites them in an elegant and simple framework.

My inquiry has proceeded from the general to the specific: I used insights from the *general* debate on nudging to model a theory for the more *specific* subject of nudging people into medical consent. But there is also room to proceed in the opposite direction, namely to ask whether my specific conclusions concerning nudging people into medical consent could also inform the permissible use of nudging more generally and even apply to contexts where consent does not play a role. As a matter of fact, I think this is possible. In these other areas too, transparency may only be a test for justification and not a self-standing condition, different ways of explicating and attacking rationality remain relevant, and easy resistibility may not always preclude illegitimate control. And since these claims motivated the idea of interpersonal justification, the permissibility of nudging in general may, at least partly, be a matter of interpersonal justification too. Yet, I elaborated on the idea of interpersonal justification in terms of the principles fundamental to the medical context and as far as medical consent is concerned. Thus, if my view is to be applied more generally, these patient-physician principles will need to be substituted by something else, i.e. principles governing the state-citizen relationship in matters of states nudging their citizens; and if my account is to be transferred to other contexts of consent, these principles might need to be different again, e.g. in an economic context, principles governing the relation between contractual parties. I cannot explore these other principles in any meaningful detail here. Yet, my approach to medical consent suggests these routes for further research and thereby also proves to be pertinent to the wider debate on nudging too.

## Footnotes

1 To clarify: in variant 2, the nurse still exploits Betty’s irrationality because he still triggers Betty’s confirmation bias: Betty considers the medical evidence *relevant* to her decision about whether to consent *only because* this white man belonging to her religious community told her so. As a result, there is still an important part in Betty’s decision-making that is procedurally irrational: selecting evidence in the way Betty does is in no way a reliable procedure when making medical decisions.

2 See for instance Noggle on the use of ‘salience nudges.’ See (Noggle, 2018).

3 It is worth noting that some people explicitly explained how some techniques of manipulation work very transparently while still reducing the quality of their target’s decision-making (Barnhill, 2014; Gorin, 2014a, 2014b).

4 In analysing the validity of consent transactions, Pufendorf focuses on the recipient of consent and says that consent is invalid if there is something “quod ineptum ipsum reddit ad ius acquirendum” (Pufendorf, 1998: Liber III Cap VI: §X. Pufendorf then affirms this view in § XI), i.e. if there is something that “disqualifies him as a recipient of a right.” (My translation.)

5 I owe this distinction to Thomas Douglas, *unpublished manuscript.*

6 For a related claim, see (Wertheimer, 2013).

7 I discuss related claims on pressure and consent in the context of living organ donation in (Kiener, 2020).

8 Thanks to an anonymous reviewer for this example.

9 This case has been reported as a true event: https://www.boredpanda.com/pediatrician-anti-vaccination-parent-conspiracy-theory/?utm_source=google&utm_medium=organic&utm_campaign=organic I simplified this case by focusing on the woman consenting to her own vaccination rather than deciding whether her child should be vaccinated.

10 Here, the physician’s influence may not only negate the voluntariness of consent but also undermine *informed* consent.

11 To clarify, a cooling-off period, as I understand it here, is the period *before* a person actually gives consent. In order to avoid confusion, it is worth pointing out that such an understanding diverges from some other contexts where a cooling-off period means the period *after* a person has consented but still retains the right to cancel a contract without penalty.

12 So understood, my view bears some resemblances to another account in the wider literature on consent, namely Miller and Wertheimer’s *fair transaction view,* according to which “A is morally permitted to proceed on the basis of a consent transaction if A has treated B fairly and responds in a reasonable manner to B’s token or expression of consent or what A reasonably believes is B’s token or expression of consent” (Miller & Wertheimer, 2010, p. 81). Like Miller and Wertheimer, I also endorse a *transactional* view, in the sense of focusing on the interaction between the person giving consent and the person receiving consent. Yet, there are two important differences. Firstly, whereas Miller and Wertheimer reject the concept of ‘valid consent’ to explain the situation where a person receives permission to do something that he could not otherwise do, my account operates within the concept of valid consent. Secondly, whereas Miller and Wertheimer admit that accounting for the intricacies of consent transactions in specific contexts “requires a more complete theory of fair transactions than we are prepared to offer here” (Miller & Wertheimer, 2010, pp. 87-88) and keep their view at a very general level, my own view offers an elaborate answer for the medical context and thereby pursues a much more specific inquiry. It is also here that my own specific emphasis on ‘interpersonal justification’ contrasts with Miller and Wertheimer’s much wider notion of ‘fairness’.
